# Retinal Structural and Microvascular Alterations in Different Acute Ischemic Stroke Subtypes

**DOI:** 10.1155/2020/8850309

**Published:** 2020-12-09

**Authors:** Ying Zhang, Ce Shi, Yihong Chen, Weicheng Wang, Shenghai Huang, Zhao Han, Xianda Lin, Fan Lu, Meixiao Shen

**Affiliations:** ^1^School of Ophthalmology and Optometry, Wenzhou Medical College, Wenzhou, Zhejiang, China; ^2^The Second Affiliated Hospital & Yuying Children's Hospital of Wenzhou Medical University, 109 Xueyuan Road, Wenzhou, Zhejiang, China

## Abstract

**Introduction:**

Retinal structural and microvascular damages reflect damage to cerebral microvasculature and neurons. We aimed to investigate neovascular unit abnormalities among patients with large-artery atherosclerosis (LAA) or small-vessel occlusion (SAA) and control subjects.

**Methods:**

Twenty-eight LAA patients, forty-one SAA patients, and sixty-five age- and gender-matched controls were recruited. Based on optical coherence tomography angiography (OCTA), retinal capillary vessel density was assessed in the general and local sectors, and the thickness of individual retinal layer was extracted from retinal structural images. The differences between structural and microvascular were analyzed.

**Results:**

The superior peripapillary retinal nerve fiber layer (pRNFL) thickness was significantly different among the three groups, and the LAA group had the thinnest thickness. Compared to the control group, the deep retinal capillary vessel density in other two stroke subgroups were significantly reduced in all regions except in the inferior region (*P* < 0.05), and the fractal dimension in C2 and C4 regions of deep retina was significantly lower in the LAA group (*P* < 0.05). *Discussion.* Compared with superficial microvascular network, deep microvascular network is more sensitive to ischemic stroke. In addition, we have demonstrated quadrant-specific pRNFL abnormalities in LAA and SAA patients. Superior quadrant pRNFL thickness differences between stroke subgroups may suggest that changes in retinal nerve fiber layer are more sensitive to subtype identification than changes in retinal microvascular structure. All in all, the alteration in retinal structural and microvascular may further elucidate the role of the neovascular unit in ischemic stroke, suggesting that the combination of these two indicators could be used for subtype identification to guide prognosis and establish a risk prediction model.

## 1. Introduction

Stroke is the most common cause of serious disability in adults, and China bears the biggest burden globally [1]. As therapeutic options are limited, effective preventive strategies for early diagnosis are needed. The underlying subclinical pathologic process occurs much earlier before the onset of clinical stroke while current neuroimaging technologies may not be capable of directly observing subtle subclinical changes in stroke due to the resolution. Besides, current predictions of stroke are difficult to quantify. Therefore, there is an urgent need for additional surrogate techniques to detect the subtle changes in vivo. In addition to the importance of finding indicators to establish a model for stroke risk prediction and prognosis assessment, the identification of subtypes of ischemic stroke is vital for guiding clinical treatment and management. However, the current international classification Treatment of Acute Stroke Trial (TOAST) [2] requires a lot of auxiliary examinations which are expensive and time-consuming. The above shortcomings and possible examination contraindications make it challenging for patients to complete the examination in time when they are admitted to the hospital, affecting the early guiding role in clinical treatment. Therefore, finding an early, especially sensitive and effective, method is of great importance in disease prediction, treatment, and prognosis assessment. Because the retinal and cerebral vessels share similar anatomic, embryological, and physiological characteristics, the retina provides a unique “window” to assess the cerebral microvascular and neurons in vivo noninvasively.

Previous studies based on fundus photography have revealed an independent correlation between retinal vascular parameters and stroke [3–6]. Additionally, several studies also detected vascular changes vary according to stroke subtypes, suggesting the specific cerebral microvasculopathy of subtyping of stroke [7, 8]. Inconsistently, another study showed that vascular changes were similar between stroke subtypes [9]. The reason for the discordant results may be that the fundus photography is only a plane picture, which only reflects the large blood vessels of the retina without microvascular and quantitative retinal structural parameters. With the advancement of the technique, the optical coherence tomography angiography (OCTA) can reflect finer retinal capillary plexuses and choriocapillaris changes by generating three-dimensional images based on the comparisons of the motion of circulating blood cells. Consequently, we can observe retinal microvascular changes of stroke patients.

The concept of the neurovascular unit (NVU) was proposed in 2003 [10], which is composed of endothelial cells, neurons, astrocytes, and pericytes. The NVU provides new insights for the pathogenesis and diagnostic and treatment strategies of stroke [11, 12]. Spectral-domain optical coherence tomography (SD-OCT) with high-resolution retinal imaging can provide cross-sectional images of biologic structures and quantify the thickness of each retinal layer. One previous study observed that the transneuronal retrograde degeneration (TRD) of retinal ganglion cells (RGCs) assessed by SD-OCT is associated with cerebral infarction [13]. Most of the studies containing previous stroke patients with an increased risk of confounding factors concentrated on the association between large retinal vessels or neural structures changes and stroke separately [3, 13]. So far, there have been no in vivo studies on simultaneous microvascular and neural structures in stroke subjects.

In this study, we aimed to find the retinal microvascular and microstructural changes in the subtypes of initial acute stroke patients.

## 2. Methods

### 2.1. Study Population

In this study, a total of 85 patients with initial ischemic stroke within 14 days of an acute period were prospectively recruited from the neurology unit at the Second Affiliated Hospital & Yuying Children's Hospital of Wenzhou Medical University from Jan 2017 to December 2018. One neurologist (Zhao Han) assessed the stroke severity with the National Institutes of Health Stroke Scale (NIHSS) [14] and classified large-artery atherosclerosis (LAA) and small-artery occlusion lacunar (SAA) according to a modified TOAST classification [2]. Besides, 65 age- and gender-matched controls with no self-reported history of stroke or transient ischemic attack or ophthalmic disease were enrolled consecutively from the relatives of patients or working staff at the Eye Hospital or the Second Affiliated Hospital & Yuying Children's Hospital of Wenzhou Medical University between Jan 2017 to Aug 2019. Considering the effect of stroke site, parameters of ipsilateral eyes were selected for analysis. Additionally, random eyes were selected in nonunilateral stroke and control subjects. Written informed consent was obtained from patients or their next of kin, and the project was approved by the ethics committee of the Eye Hospital of Wenzhou Medical of Wenzhou Medical University.

### 2.2. Assessment of Cardiovascular Risk Factors

Patients finished detailed questionnaires for information on history of hypertension, diabetes mellitus, hypercholesterolemia, ischemic heart disease, cigarette smoking status, and medication use. All patients underwent usual examinations of stroke, including brain imaging, fasting blood samples for glycosylated hemoglobinA1C (HbA1C), total cholesterol (TC), total triglycerides (TG), homocysteine (HCY), creatinine (Cr), high-density lipoprotein (HDL-C), low-density lipoprotein (LDL-C), and body mass index (BMI). Besides, as a part of clinical care for stroke, blood pressure was measured three times after participants had been seated for at least 10 minutes at the same sitting. The mean blood pressure of three times was taken as the final result. The mean arterial pressure (MAP) is equal to one-third of systolic blood pressure (SBP) plus two-thirds of diastolic blood pressure (DBP).

Hypertension was diagnosed as SBP ≥140 mm Hg or DBP ≥90 mm Hg at examination or a self-reported history of physician-diagnosed hypertension or the use of antihypertensive medication. Diabetes mellitus was defined as fasting blood glucose ≥7.0 mmol/L and/or random blood glucose ≥11.1 mmol/L, hemoglobin A1C ≥7%, self-reported history of physician-diagnosed diabetes mellitus, or the use of antihyperglycemic medication. Hypercholesterolemia was defined as fasting total cholesterol ≥5.2 mmol/L, self-reported history of physician-diagnosed hypercholesterolemia, or the use of antilipemic medication. Current smokers were defined as people who smoke currently or quitted smoking less than one year before the examination.

### 2.3. Assessment of Ophthalmic Parameters and Microstructure of the Retina

All the subjects had detailed ophthalmologic examinations performed by two ophthalmologists (Ying Zhang and Ce Shi), including slit-lamp biomicroscopy, refraction diopter, best-corrected visual acuity (BCVA), and noncontact intraocular pressure (IOP). All patients were imaged by an OCT system (Optovue RTVue-XR Avanti; Optovue, Inc., Fremont, CA, USA) to obtain the OCTA images, with detailed steps described as follows: refraction data were converted to spherical equivalents (SEs) and calculated as the spherical dioptric power plus one-half of the cylindrical dioptric power. The exclusion criteria were presented as follows: patients with contraindications to magnetic resonance, hemorrhagic stroke, recurrent stroke, those who were unable to complete the eye examinations, and those with spherical equivalent (SE) under ± 5.00 *D*, IOP >21 mm Hg or previous ophthalmologic diseases (such as cataract, glaucoma, high myopia, and retinal diseases). Other exclusion criteria were systemic diseases that could affect the ocular structures, such as uncontrolled hypertension/diabetes and neurological diseases such as Parkinson's disease and multiple sclerosis.

### 2.4. MRI Analysis

All patients underwent the 3.0-T MRI (Signa HDxt GE Healthcare), which included T1-weighted and T2-weighted imaging, diffusion weighted imaging (DWI), and fluid attenuated inversion recovery (FLAIR). The slice thickness was 5 mm with an interslice gap of 1 mm. The high signal on DWI sequence of MRI indicates the presence of acute cerebral infarction. In addition, the size and the location of the lesion are conductive to the classification of stroke. In the control group, MRI was also used for the reason of homogeneous management. For ethical reasons, not all controls were willing to undergo the tests for fasting blood tests and magnetic resonance imaging. Therefore, the two indicators are not shown in the Table 1.

### 2.5. OCT and OCTA Acquisitions

All subjects remained seated under the same conditions, and examinations were performed by an expert examiner. The OCTA system, which employs the split-spectrum amplitude-decorrelation angiography (SSADA) algorithm, operated at a rate of 70000 A-scans per second, with the scan area of 3 × 3 mm^2^, and the results were obtained by orthogonal registration and merging of two consecutive B-scans. The size of the exported OCT images was 304 × 304 pixels. OCTA combines orthogonal fast-scan directions to correct motion artifacts based on the DualTrac Motion Correction technology [15]. A good set of scans with a signal strength index (SSI) over 40 was selected for further analysis.

#### 2.5.1. Retinal Layer Thickness Analysis on Spectral-Domain Optical Coherence Tomography

**Figure 1:**
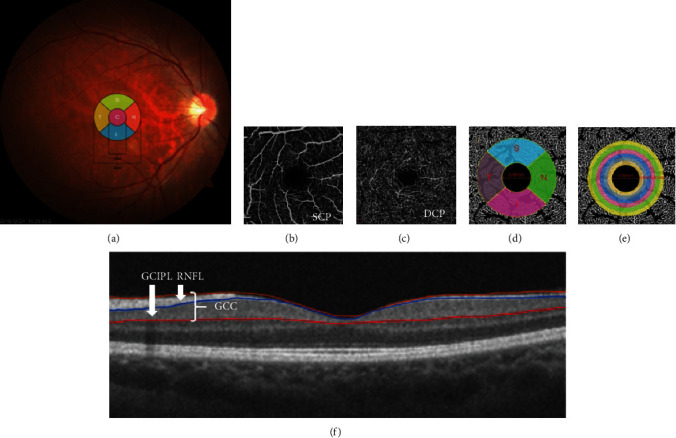
Retinal structure and the corresponding microvascular density and fractal dimension in superficial and deep layers imaged by OCT/OCTA. (a) Five regions within a 3 mm area around the fovea: (C) central region; (S) superior; (T) temporal; (I) inferior; (N) nasal. (b, c) OCTA images of superficial capillary plexuses (SCP) and deep capillary plexuses (DCP) in 3 × 3 mm area around fovea. (d, e) Fractal dimension analysis in four quadrants and six annular zones excluding the avascular zone (diameter = 0.6 mm): C1 (diameter = 0.92 mm), C2 (diameter = 1.23 mm), C3 (diameter = 1.55 mm), C4 (diameter = 1.87 mm), C5 (diameter = 2.18 mm), and C6 (diameter = 2.50 mm). (f) Retinal nerve fiber layer (RNFL) and ganglion cell complex (GCC) thickness around the fovea. GCIPL = ganglion cell-inner plexiform layer.

Retinal thickness was imaged by the RTVue XR Avanti SD-OCT system (Optovue, Inc, Fremont, California, USA). Besides, the average, superior (S), temporal (T), inferior (I), and nasal (N) quadrants of peripapillary retinal nerve fiber layer (pRNFL) thickness were obtained. The ganglion cell complex (GCC) provides inner retinal thickness values from the internal limiting membrane (ILM) to the inner molecular layer (IPL), shown as average, superior, and inferior regions (Figure 1(f)).

#### 2.5.2. Capillary Vessel Density and Fractal Dimension Analysis on Optical Coherence Tomography Angiography

Retinal microvasculature was evaluated by the RTVue XR with AngioVue (software version 2017.1.0.155; Optovue, Inc, Fremont, CA, USA). Patients underwent different types of scanning: 3 × 3 mm^2^ angioretina around the fovea, 4.5 × 4.5 mm^2^ angioretina scans around the optic nerve head (ONH), 3D retina scans, retinal map scans, and radial lines scans.

Vessel density (VD) is defined as the percentage of area occupied by OCTA detected vasculature. The software sets the superficial capillary plexuses (SCP) from 3 *μ*m below the ILM to 15 *μ*m below the IPL. The deep capillary plexuses (DCP) were set from 15 to 70 *μ*m below the IPL (Figures 1(b) and 1(c)). In addition, the parafovea vessel density, defined as the area of annular circle with a diameter of 3 mm excluding the fovea zone (diameter = 1 mm), was divided automatically into whole and superior (S), temporal (T), inferior (I), and nasal (N) quadrants. Similarly, the 5 sectors of radial peripapillary capillary (RPC) vessel density were analyzed. The boundary of RPC ranges from ILM to the nerve fiber layer.

To quantify the complexity of the branching pattern and density of the retinal capillary network in OCTA images, the automated fractal analysis system was employed to correct the image magnification based on the axial length [16, 17]. Briefly, the OCTA images in PNG format were imported to the custom automated algorithm software published previously [18]. Then, the grayscale of the two-dimensional OCTA images was first extended by bicubic interpolation to 1024 × 1024 pixels so as to improve the image details. The binary images of vessels were created by the algorithm. Subsequently, one binary image containing only large arteries and the other binary image containing both large and small vessels were subtracted to obtain the final binary image. Based on the final image of white-pixelated vasculature, a skeletonized image was created by detecting the central axis of each capillary. After the image processing, both the superficial and deep retinal capillary complexities were calculated based on the skeletonized images [19, 20]. The quantitative measured parameter of complexity, D_box_ values, was obtained with the fractal analysis software (Benoit, Trusoft Benoit Fractal Analysis Toolbox; Trusoft International, Inc., St. Petersburg, FL). Both the general and local fractal dimensions were used to describe the complexity of capillary network. At first, after excluding the fovea avascular zone (FAZ) within the diameter of 0.6 mm, the fractal dimension (FD) was automatically calculated for the total annular zone (TAZ) within the 2.5 diameter and for the 4 parafoveal quadrant sectors (superior (S), temporal (T), inferior (I), and nasal (N)) and 6 concentric isometric annular rings (Figures 1(d) and 1(e)). The methods above were implemented using MATLAB v 7.10 (Mathworks, Inc., Natick, Massachusetts, USA).

### 2.6. Statistical Analysis

All statistical analyses were conducted using SPSS software (version 24.0; SPSS, Inc., Chicago, IL, USA). The data were expressed as the mean ± standard deviation (SD). One-way analysis of variance (ANOVA) was used to test the differences among patients with large-artery stroke and lacunar stroke and control subjects, and Bonferroni correction was used for pairwise comparisons. The differences in gender and medical history were determined by the *χ*^2^ test.

## 3. Results

A total of 85 patients were included in the present study. Among them, 16 patients were excluded due to poor image quality of OCTA scans. The remaining 28 patients with LAA, 41 patients with SAA, and 65 age-and gender-matched control subjects were further analyzed. The demographic and clinical characteristics are summarized in Table 1. Normally, distributed data are represented by mean plus or minus standard deviation, while nonnormally distributed data are represented by median and interquartile spacing. Differences in age, sex, BMI, SE, IOP, and DBP, together with the prevalence of hyperlipidemia, currently smoking, and previous ischemic heart disease were not significant among the three groups. Patients with SAA were more likely to have hypertension than the other two groups (*P* < 0.001). Besides, the values of SBP and MAP were significantly higher than those of the other two groups (*P* < 0.001). LAA patients were more likely to undergo diabetes (*P* = 0.007) than the controls. However, there was no statistical difference in demographic data between the two stroke subgroups. Not all subjects completed all tests. The number of eyes that completed each examination is shown in Table 2.

### 3.1. Intergroup Differences among the Three Groups

#### 3.1.1. Retinal Microstructural Thicknesses

**Figure 2:**
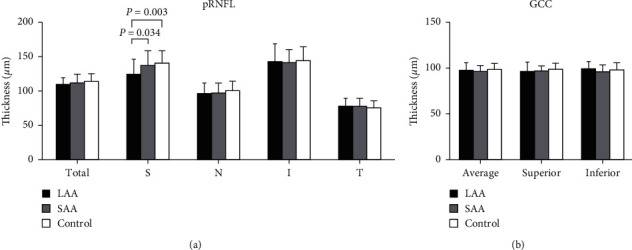
(a) Peripapillary retinal nerve fiber layer (pRNFL) thickness in eyes of patients with large-artery atherosclerosis (LAA), small-vessel occlusion (SAA), and controls. *S* = superior; *N* = nasal; I = inferior; *T* = temporal. (b) Ganglion cell complex (GCC) thickness in three groups.

In terms of the quadrants, the superior pRNFL thickness was significantly thinner in the eyes of patients than that in the eyes of the control group (*P* = 0.01, Table 3, Figure 2(a)). In the eyes of LAA, the pRNFL thickness was significantly thinner in the superior quadrant compared to the eyes of SAA (*P* = 0.034) and control (*P* = 0.003). No significant superior pRNFL thinning was observed in SAA compared to control (*P* = 0.438). Additionally, the thicknesses of GCC were not significantly different among the three groups (all *P* > 0.05, Table 3, Figure 2(b)).

#### 3.1.2. Vessel Density around ONH and Macula

**Figure 3:**
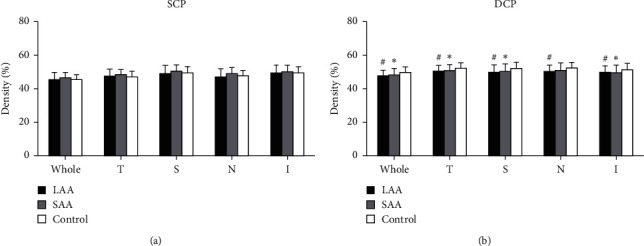
Comparisons of the microvascular density on OCTA images in whole and four quadrant sectors of the superficial capillary plexuses (SCP) (a) and deep capillary plexuses (DCP) (b). ^*∗*^*P* < 0.05, the density in the SAA group was lower than that in the control group; ^#^*P* < 0.05, the density in the LAA group was lower than that in the control group.

The vessel density around the macula in the deep retinal capillary layer was significantly reduced in patients with LAA or SAA within all regions, except for the inferior region (*P* < 0.05, Table 4, Figure 3(b)). The significant differences mostly existed between the stroke group and control group (*P* < 0.05), and no significant difference was found in the two stroke subgroups, although the LAA group tended to have a lower vessel density. In addition to ONH capillary density, no significant difference was found in the superficial layer of all regions (*P* > 0.05, Table 4, Figure 3(a)).

#### 3.1.3. Fractal Dimension around Macula

**Figure 4:**
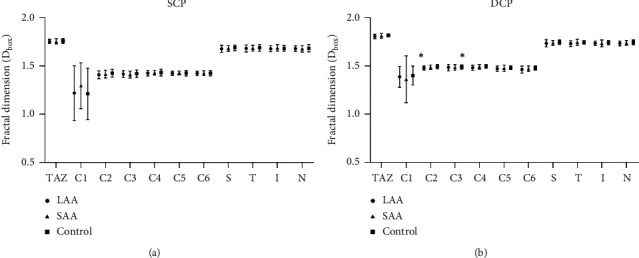
Comparisons of the microvascular D_box_ among patients with large-artery atherosclerosis (LAA), small-vessel occlusion (SAA), and controls. Comparisons in (a) superficial capillary plexuses (SCP) and (b) deep capillary plexuses (DCP). A significant reduction was seen in the deep layer of C2 and C4 region in LAA patients when compared to controls. ^*∗*^*P* < 0.05.

Differences in the fractal dimension were only statistically significant in C2 and C4 regions of the deep retina (*P* < 0.05, Table 5, Figure 4(b)) between patients with LAA and the controls. Compared with the control group, the fractal dimension of most regions in the stroke group showed a downward trend, although there was no statistical difference in most regions (*P* > 0.05, Table 5, Figures 4(a) and 4(b)).

## 4. Discussion

Assuming that the retinal vasculature mirrors the cerebral vasculature, OCTA enables noninvasive imaging of retinal capillaries in multiple layers invisible on fundus images. Based on fundus photography, retinal abnormalities, including arteriovenous nicking and generalized and localized arteriolar thinning, a lower arteriolar/venular diameter ratio and geometric parameters have been demonstrated to be significantly related to the incidence of stroke [5, 9, 21, 22]. The previous studies using fundus photos could not qualitatively detect the subtle changes at the capillary level while OCTA provides the opportunity to investigate the retinal capillary microcirculation at the micrometer resolution [23].

In terms of vessel density around the macula, our findings demonstrate that the changes of vessel density are more obvious in the deep layers than the superficial layer between stroke patients and control subjects. Our results point to a preferential involvement of the deep layer in patients with stroke which may be attributed to the fact that the deep network consists of a dense and complex system of smaller vessels [24]. It can be speculated that deep retinal capillaries are more susceptible to ischemic and hypoxia. As per our expectation, patients with SAA displayed much smaller changes than patients with LAA. However, there exists no significant difference between the LAA and SAA. The finding suggested that the retinal vasculopathy may result from downstream effects of large-artery pathology in the cerebral circulation. In addition, we found that fractal dimension was not helpful in identifying stroke subtypes and was significantly lower in the LAA group than in controls only in individual regions of the deep retina. Our results were in consistence with those of others [9], which found that decreased FD was correlated with stroke, suggesting a loss of complexity. However, previous results of fractal dimension based on the fundus photograph remain controversial. Some considered lacunar stroke subtype was associated with decreased retinal FD [8] while others demonstrated that the lacunar stroke was positively associated with higher FD [7]. We found no significant difference between stroke subgroups, which may indicate that the FD is not applicable to differentiate the disease subgroups who have already had stroke.

Regarding peripapillary vessel density, there was no significance in RPC among the groups. The finding could be related to the anatomical differences between the different areas. The parafoveal superficial capillary plexus originates largely from the retinal circulation, whereas the RPC receives additional blood supply from the choroid [25]. Larger vascular channels around the optic disc may have masked subtle changes in the capillary network. Additionally, RPC contains multiple layers of capillaries that overlap on en-face OCTA images, lacking the ability to detect tiny vascular losses.

In addition to retinal capillary changes, our study observed that the pRNFL thickness was statistically reduced in the superior quadrant of stroke patients, and there was a statistical difference between the stroke subgroups which may indicate different patterns of nerve damage in the two stroke subtypes. Additionally, some research recently also observed that both acute and previous stroke were significantly associated with retinal nerve fiber layer defects (RNFLDs) [13]. These findings were also in accordance those of with others [26], which reported the associations between severity and laterality of RNFLD and laterality of hemispheric damage as well as arterial territory of infarct. They found RNFLDs were significant in the temporal sector of the ipsilateral side and in the nasal sector of the contralateral side of the stroke. Furthermore, they also confirmed that the degree of the transneuronal retrograde degeneration (TRD) was time-dependent. However, we find that the significant RNFLDs only exist in the superior sector in our study although the ipsilateral sides were included.

As is known to all, over 30 morphological types of RGCs compose the structure of the retina. The midget RGCs (80%), with wide retinal dendritic fields located in the peripheral retina, mainly project to the magnocellular layers of the lateral geniculate body. The parasol RGCs (5–15%) predominantly present in the papillomacular bundle (macula) and project to suprachiasmatic nucleus of the hypothalamus. Axons from midget RGCs enter the superior, inferior, and nasal sectors of the optic nerve, and parasol RGCs gather their axons and enter the temporal sector of the optic nerve [27, 28]. The different degenerative patterns of the ganglion cells and nerve fibers have been demonstrated in several neurodegenerative diseases [29, 30]. Recent studies have described that the parasol RGCs are more involved in the pathogenesis of Alzheimer's disease [31–33]. However, as for the Parkinson syndrome and mitochondrial optic neuropathy, the midget RGCs are mainly involved [30, 34, 35]. Both the above and our study suggest that mean thickness measurement may not reflect the disease well and reduce the diagnostic efficacy of the ocular biomarker. We speculate that detailed analysis of the focal nerve structure alteration may be developed as an ocular imaging biomarker for monitoring disease progression and evaluating prognosis of these diseases.

Nevertheless, as this is the first study on ischemic stroke subtypes based on OCTA, further large samples are required to confirm the universality in the disease. The discrepancies existing between different studies could be ascribed to differences in course and severity of disease as well as various OCT devices and study designs.

Besides, a previous animal study also revealed that the TRD of retinal ganglion cells occurred after shrinkage of the optic tract, and degeneration of the RGC was progressing slowly in the next few years [36]. Thus, our negative results in GCC thickness might be related to the fact that patients were tested within two weeks after stroke.

To conclude, our study provides a side view that the neurovascular unit is affected in ischemic stroke and severely in LAA patients. The components of neurovascular units are interrelated in the microenvironment. Studies have shown that the signal transmission between neurons, astrocytes, and microvascular endothelial cells regulates the brain microenvironment [37] [29]. Briefly, NVU as a structural and functional whole, the relationship between members changes in the state of illness. The mechanism of cerebral ischemia is complex and involves multiple cascading reactions. Therefore, monitoring the NVU as a whole and improving its function help maintain brain cell function and make stroke treatment more ideal. The retina provides a visualization of the neurovascular units simultaneously to reflect the changes of the brain, which has important implications for disease surveillance.

In addition, we also acknowledge the limits in this study. The cross-sectional study with a small sample limits our ability to identify the different pathogenesis of the capillaries and microstructures of the stroke. The outer structure of the retina which is mainly supplied by the choroid has not been analyzed. Due to the practical difficulty of recruiting and examining the patients, milder patients may be recruited, which may be more likely with milder vascular lesions to differentiate the two subtypes. Despite the weakness, there are several strengths in our study. We recruited different ischemic stroke subtypes with strict inclusion and exclusion criteria. Otherwise, to minimize the confounding of stroke, the patients with a history of previous stroke were excluded. We completed the detailed ophthalmic and clinical examinations within two weeks after the onset of the stroke and maintained the blinding of retinal and brain images to each other. Finally, this is the first attempt to simultaneously observe retinal microvascular and neurological changes in different stroke subtypes in vivo.

Moreover, further longitudinal studies with a larger sample size are required to show differences in the characteristics of retinal microstructure and capillaries. It remains to be seen whether the retinal signs are indicative of cerebrovascular risk beyond the conventional risk indicators and whether the retinal imaging technique will be a surrogate or accessory examination system in clinical settings and ultimately become a part of the routine stroke risk or evaluation of treatment assessment.

## Figures and Tables

**Table 1:** Demographic characteristics of study subjects.

	LAA	SAA	Control	*P* ^*∗*^	*P*1	*P*2	*P*3
Eyes,	28	41	65	—	—	—	—
Age, *y*, mean ± SD	60.07 ± 10.10	58.83 ± 7.72	59.00 ± 7.20	0.797	0.529	0.557	0.915
Sex, female(%)	8 (28.6)	11 (26.8)	27 (41.5)	0.231	0.874	0.236	0.124
BMI	24.90 ± 2.97	24.53 ± 2.87	25.08 ± 2.38	0.596	0.580	0.776	0.310
SE, diopter	0.43 ± 1.33	0.38 ± 1.18	0.24 ± 1.06	0.741	0.860	0.497	0.567
BCVA, LogMAR	0.02 ± 0.12	0.01 ± 0.10	-0.03 ± 0.08	0.024	0.619	0.018	0.037
AL, mm	23.30 ± 0.95	23.37 ± 0.89	23.14 ± 0.91	0.550	0.777	0.528	0.294
IOP, mm Hg	12.05 ± 2.56	12.68 ± 3.19	12.15 ± 2.53	0.559	0.363	0.877	0.341
SBP, mm Hg	151.61 ± 21.85	155.22 ± 17.15	134.37 ± 14.79	<0.001	0.393	<0.001	<0.001
DBP, mm Hg	87.64 ± 13.28	90.61 ± 12.92	85.80 ± 11.89	0.160	0.335	0.516	0.056
MAP, mm Hg	108.96 ± 14.05	112.15 ± 12.53	101.99 ± 11.50	<0.001	0.296	0.014	<0.001
Hypertension, *n*(%)	21 (75.0)	35 (85.4)	23 (35.4)	<0.001	0.280	<0.001	<0.001
Hyperlipidemia, *n*(%)	9 (32.1)	5 (12.2)	17 (26.2)	0.112	0.043	0.555	0.084
Current smoker, *n*(%)	16 (57.1)	21 (51.2)	24 (36.9)	0.135	0.628	0.071	0.147
Diabetes, *n*(%)	7 (25.0)	5 (12.2)	3 (4.6)	0.016	0.205	0.007	0.256
Previous ischemic heart disease, *n*(%)	2 (7.1)	3 (7.3)	2 (3.1)	0.527	>0.999	0.581	0.373
HbA1c, (%)	5.95 (5.53–6.28)	5.80 (5.55–6.20)	—	0.433	—	—	—
TG, mmol/L	1.99 (1.30–2.97)	1.58 (1.18–1.87)	—	0.051	—	—	—
TC, mmol/L	4.81 ± 0.96	4.48 ± 0.76	—	0.117	—	—	—
HDL-C, mmol/L	2.78 (0.80–1.13)	0.95 (0.81–1.13)	—	0.898	—	—	—
LDL-C, mmol/L	2.73 ± 0.68	2.57 ± 0.76	—	0.353	—	—	—
HCY, *μ*mol/L	10.60 (8.75–15.20)	10.20 (8.98–13.90)	—	0.684	—	—	—
Cr, *μ*mol/L	65.65 (57.90–77.83)	68.50 (58.50–81.73)	—	0.501	—	—	—
NIHSS score, mean ± SD (range)	2 (1–3)	1 (1–2)	—	0.097	—	—	—

LAA, large-artery atherosclerosis; SAA, small-vessel occlusion; BMI, body mass index; SE, spherical equivalent; BCVA, best-corrected visual acuity; AL, axial length; IOP, noncontact intraocular pressure; SBP, systolic blood pressure; DBP, diastolic blood pressure; MAP, mean arterial pressure; HbA1c, hemoglobin; TG, total triglycerides; TC, total cholesterol; HDL-C, high-density lipoprotein; LDL-C, low-density lipoprotein; HCY, homocysteine; Cr, creatinine; NIHSS, NIH Stroke Scale; M, male; F, female; ^*∗*^*P* value among the three groups; *P*1 and *P* values between LAA and SAA; *P*2 and *P* values between LAA and control; and *P*2 value between SAA and control.

**Table 2:** The numbers of eyes examined in all three subgroups.

	Age	BMI	MAP	SE	BCVA	IOP	AL	RPC	pRNFL	GCC	Superficial VD	Deep VD	FAZ	Superficial FD	Deep FD
LAA	28	26	28	25	27	27	20	17	17	26	27	28	28	28	28
SAA	41	39	41	40	40	40	28	27	27	34	39	40	39	41	41
Control	65	64	65	64	65	65	47	63	63	64	64	65	65	65	65

LAA, large-artery atherosclerosis; SAA, small-vessel occlusion; BMI, body mass index; MAP, mean arterial pressure; SE, spherical equivalent; BCVA, best corrected visual acuity; IOP, intraocular pressure; AL, axial length; RPC, radial peripapillary capillary; pRNFL, peripapillary retinal nerve fiber layer; GCC, ganglion cell complex; VD, vessel density; FAZ, fovea avascular zone; FD, fractal dimension.

**Table 3:** Retinal thickness (*μ*m) from commercial instruments in patients with LAA, SAA, and controls.

Layers	Regions	LAA	SAA	Control	*P* ^*∗*^	*P*1	*P*2	*P*3
pRNFL	Total	109.35 ± 10.06	111.85 ± 12.54	114.19 ± 10.86	0.254	0.472	0.117	0.366
	S	124.65 ± 21.47	137.41 ± 21.07	140.84 ± 17.68	0.010	0.034	0.003	0.438
	N	96.65 ± 14.85	96.93 ± 14.68	100.27 ± 14.01	0.472	0.950	0.356	0.312
	I	143.25 ± 25.41	141.56 ± 18.58	144.40 ± 19.75	0.832	0.793	0.841	0.546
	T	78.47 ± 10.54	77.52 ± 11.78	75.35 ± 10.21	0.468	0.774	0.289	0.382

GCC	Average	98.23 ± 7.53	96.50 ± 6.18	98.55 ± 6.66	0.348	0.325	0.840	0.154
	Superior	96.77 ± 9.84	97.03 ± 5.58	98.92 ± 6.50	0.292	0.888	0.195	0.212
	Inferior	99.81 ± 7.24	96.09 ± 7.41	98.20 ± 7.67	0.158	0.060	0.360	0.187

LAA, large-artery atherosclerosis; SAA, small-vessel occlusion; pRNFL, peripapillary retinal nerve fiber layer; GCC, ganglion cell complex. S = superior; N = nasal; I = inferior; T = temporal; ^*∗*^*P* value among the three groups; *P*1 and *P* values between LAA and SAA; *P*2 and *P* values between LAA and control; *P*3 value between SAA and control.

**Table 4:** Comparison of microvascular density in patients with LAA, SAA, and controls between the superficial and deep retinal capillary plexus.

Layers	Regions	LAA	SAA	Control	*P* ^*∗*^	*P*1	*P*2	*P*3
RPC	Whole	49.51 ± 2.54	49.47 ± 3.14	49.98 ± 2.28	0.621	0.964	0.504	0.393
	S	52.94 ± 5.87	53.22 ± 4.48	53.02 ± 3.86	0.973	0.836	0.950	0.838
	N	49.00 ± 5.35	50.63 ± 5.83	51.79 ± 5.24	0.155	0.333	0.062	0.352
	I	55.56 ± 3.65	53.11 ± 4.37	53.76 ± 4.21	0.174	0.066	0.127	0.500
	T	53.00 ± 5.23	51.07 ± 6.07	52.95 ± 5.29	0.308	0.260	0.974	0.141

SCP	Whole	45.59 ± 4.26	46.72 ± 3.13	45.65 ± 2.82	0.209	0.478	0.998	0.196
	T	47.72 ± 4.06	48.50 ± 3.01	47.15 ± 3.37	0.157	0.365	0.471	0.055
	S	49.23 ± 4.82	50.64 ± 3.63	49.56 ± 3.67	0.277	0.154	0.717	0.178
	N	47.18 ± 4.76	49.15 ± 3.48	47.91 ± 3.10	0.107	0.169	0.743	0.169
	I	49.62 ± 4.60	50.30 ± 3.74	49.58 ± 3.59	0.631	0.481	0.967	0.360

DCP	Whole	47.49 ± 3.12	48.11 ± 3.70	49.46 ± 3.14	0.017	0.448	0.009	0.044
	T	50.30 ± 3.17	50.58 ± 3.55	52.04 ± 3.05	0.020	0.730	0.018	0.025
	S	49.62 ± 4.23	50.20 ± 4.24	51.77 ± 3.67	0.029	0.556	0.018	0.050
	N	50.26 ± 3.46	50.67 ± 4.37	52.19 ± 3.17	0.027	0.645	0.020	0.039
	I	49.58 ± 3.65	49.31 ± 4.46	51.09 ± 3.67	0.051	0.783	0.090	0.026

LAA, large-artery atherosclerosis; SAA, small-vessel occlusion; RPC, radial peripapillary capillary; SCP, superficial capillary plexuses; DCP, deep capillary plexuses; S = superior; N = nasal; I = inferior; T = temporal; ^*∗*^*P* value among the three groups; *P*1 and *PP* values between LAA and SAA; *P*2 and *P* values between LAA and control; *P*3 value between SAA and control.

**Table 5:** D_box_ in the superficial and deep retinal capillary layers.

Layers	Regions	LAA	SAA	Control	*P* ^*∗*^	*P*1	*P*2	*P*3
SCP	TAZ	1.754 ± 0.020	1.756 ± 0.025	1.759 ± 0.024	0.530	0.758	0.305	0.432
	C1	1.218 ± 0.285	1.295 ± 0.238	1.210 ± 0.268	0.240	0.230	0.891	0.104
	C2	1.409 ± 0.040	1.416 ± 0.041	1.425 ± 0.041	0.224	0.490	0.098	0.301
	C3	1.415 ± 0.036	1.411 ± 0.037	1.419 ± 0.039	0.520	0.645	0.615	0.257
	C4	1.426 ± 0.029	1.430 ± 0.025	1.432 ± 0.035	0.748	0.606	0.447	0.820
	C5	1.424 ± 0.022	1.428 ± 0.020	1.425 ± 0.028	0.748	0.486	0.829	0.541
	C6	1.423 ± 0.022	1.426 ± 0.024	1.426 ± 0.028	0.857	0.633	0.601	0.996
	S	1.679 ± 0.036	1.683 ± 0.030	1.689 ± 0.028	0.316	0.559	0.149	0.356
	T	1.683 ± 0.036	1.686 ± 0.030	1.689 ± 0.035	0.745	0.681	0.448	0.722
	I	1.681 ± 0.032	1.688 ± 0.034	1.684 ± 0.030	0.607	0.325	0.609	0.527
	N	1.682 ± 0.029	1.679 ± 0.033	1.686 ± 0.038	0.670	0.739	0.675	0.376

DCP	TAZ	1.805 ± 0.023	1.810 ± 0.025	1.816 ± 0.016	0.065	0.753	0.088	0.352
	C1	1.388 ± 0.110	1.362 ± 0.244	1.401 ± 0.098	0.466	0.500	0.721	0.218
	C2	1.479 ± 0.021	1.487 ± 0.023	1.493 ± 0.023	0.019	0.118	0.005	0.204
	C3	1.484 ± 0.033	1.484 ± 0.031	1.489 ± 0.023	0.667	0.956	0.471	0.453
	C4	1.482 ± 0.026	1.490 ± 0.024	1.496 ± 0.019	0.015	0.158	0.005	0.131
	C5	1.473 ± 0.026	1.476 ± 0.035	1.483 ± 0.021	0.172	0.681	0.096	0.166
	C6	1.463 ± 0.036	1.471 ± 0.030	1.479 ± 0.022	0.067	0.622	0.097	0.321
	S	1.736 ± 0.037	1.739 ± 0.026	1.747 ± 0.021	0.126	0.708	0.077	0.121
	T	1.733 ± 0.030	1.746 ± 0.030	1.746 ± 0.018	0.062	0.037	0.029	0.923
	I	1.737 ± 0.023	1.735 ± 0.035	1.743 ± 0.023	0.244	0.779	0.277	0.116
	N	1.734 ± 0.025	1.741 ± 0.024	1.746 ± 0.023	0.098	0.271	0.034	0.285

LAA, large-artery atherosclerosis; SAA, small-vessel occlusion; SCP, superficial capillary plexuses; DCP, deep capillary plexuses; TAZ, total annular zone; ^*∗*^*P* value among the three groups; *P*1 and *P* values between LAA and SAA; *P*2 and *P* values between LAA and control; *P*3 value between SAA and control.

## Data Availability

All data generated or analyzed during this study are included within published article.
